# Patient characteristics and factors contributing to recurrence of bacterial vaginosis presented in primary care

**DOI:** 10.1093/fampra/cmad005

**Published:** 2023-01-27

**Authors:** Natasja S Delfstra, Annemarie A Uijen, M Caroline Vos, Reinier Akkermans, Antoine L Lagro-Janssen, Doreth A M Teunissen

**Affiliations:** Department of Primary Care and Community Care, Radboud University Medical Centre, Geert Grooteplein Zuid 21, 6525 EZ, Nijmegen, The Netherlands; Department of Primary Care and Community Care, Radboud University Medical Centre, Geert Grooteplein Zuid 21, 6525 EZ, Nijmegen, The Netherlands; Department of Obstetrics and Gynaecology, Elisabeth-TweeSteden Hospital, PO Box 90151, 5000 LC Tilburg, The Netherlands; Department of Primary Care and Community Care, Radboud University Medical Centre, Geert Grooteplein Zuid 21, 6525 EZ, Nijmegen, The Netherlands; Department of Primary Care and Community Care, Radboud University Medical Centre, Geert Grooteplein Zuid 21, 6525 EZ, Nijmegen, The Netherlands; Department of Primary Care and Community Care, Radboud University Medical Centre, Geert Grooteplein Zuid 21, 6525 EZ, Nijmegen, The Netherlands

**Keywords:** antibiotics, at-risk groups, electronic medical records, primary care, women’s health/gynecology

## Abstract

**Background:**

Bacterial vaginosis (BV) is a common problem in primary care. BV symptoms often have a negative impact on patients’ quality of life and may predispose to gynaecological problems. Some patients experience recurring episodes of BV. This study’s objective is to identify possible factors that may be associated with BV recurrence and describe the characteristics of these patients and interventions performed by general practitioners.

**Methods:**

In this retrospective cohort study, we used data from a primary care registration network in the Netherlands in the period 2015–2020. We analysed differences between patients with recurrent BV and patients with a single episode of BV in terms of characteristics and interventions performed by general practitioners.

**Results:**

We found that patients with recently prescribed antibiotics, and a medical history of sexually transmitted infections and/or Candidiasis significantly more often presented with recurrent BV. Patients with recurrent BV had more remote consultations and less in-person consultations than single-episode patients. The reason for encounter was more often a request for medication. Regarding GPs’ diagnostic and therapeutic interventions, microbiological tests were more frequently performed in recurrent BV patients. Moreover, most patients in both groups were prescribed oral metronidazole most frequently.

**Conclusions:**

Our findings might help GPs to better recognise patients at risk of recurrence. GPs could re-evaluate their approach to the diagnosis and treatment of recurrent BV, opting for in-person consultation and using standardised diagnostic criteria and microbiological testing in patients with recurrent complaints. Antibiotic use for other conditions in these patients may lead to new BV episodes.

**Key messages**

- Patients with recurrent bacterial vaginosis more often had remote consultations.
- Diagnostic tests were not performed in 37% of patients with recurrent BV.
- Patients with recent antibiotic use more often present with recurrent complaints.
- Re-evaluation of the diagnosis is warranted in patients with recurrent symptoms.

## Introduction

One of the most common gynaecological problems in premenopausal patients in primary care is abnormal vaginal discharge.^1^ Bacterial vaginosis (BV) is one of its possible causes. About a third of the individuals in a large population-based study were found to have a vaginal microbiome resembling BV, of which not all had complaints as only 41.6% of total participants had self-reported urogenital symptoms.^2^ However, prevalence rates in primary care are unknown.

Research has shown an association between BV and different patient factors, including country of origin,^3^ level of education,^4,5^ pessary use,^6^ and contraceptive use.^5,7^ Furthermore, BV is associated with obstetric complications and gynaecological infections, such as urinary tract infections,^8^ an increased risk of acquisition and transmission of sexually transmitted infections (STIs),^9,10^ pelvic inflammatory disease (PID),^11^ preterm birth and late miscarriage,^12,13^ and perinatal infections.^14^

BV can resolve spontaneously without treatment. Previous studies report spontaneous cure rates between 5 and 10% in non-pregnant and around 50% in pregnant patients.^15,16^ If BV does not recover spontaneously, antimicrobial therapy is indicated. Treatment of first choice is metronidazole, given either orally or intravaginally. An alternative option is intravaginally administered clindamycin.^17,18^

Despite treatment, some patients present with recurring episodes of BV. Recurrence rates can be as high as 58% over the course of 12 months.^19^ Research shows that recurrence has a negative physical and psychological impact on patients, including worries about long term complications, frustration with the lack of control over recurrence, and negative effects on self-esteem and sexual life.^20^

In spite of the considerable negative impact on patients’ quality of life and consequence of gynaecological sequelae, predisposing factors contributing to recurrence of BV have hardly been identified. Moreover, there is insufficient data on the characteristics of patients presenting with recurrent BV as well as on the interventions performed by general practitioners (GPs).

The present study aims to analyse the differences in patient characteristics and GPs’ diagnostic and therapeutic interventions in patients with one episode of BV versus patients with recurrent BV. Gaining insight into these characteristics might lead to early recognition of risk factors and, ultimately, to better therapeutic interventions in patients presenting with recurrent BV.

## Methods

### Study design

In this retrospective cohort study, data from Family Medicine Network (FaMe-Net) is used.^21^ FaMe-Net is a primary care registration network in The Netherlands including seven general practices (30 GPs and around 38,000 registered patients), in which electronic patient data is registered and coded according to the International Classification of Primary Care (ICPC) and International Classification of Diseases (ICD-10). Participating GPs have recorded every patient encounter since 1985 in an episode-of-care structure. An episode of care encompasses all encounters between GP and patient relating to a specific health problem. All forms of contact, such as via telephone, email or in-person consultation, as well as all interventions carried out by the GP during these encounters (e.g. physical examination, diagnostic tests, prescriptions, and referrals) are systematically coded. Moreover, the reason for encounter (RFE), which is the reason the patient states for seeking care, is coded. Additionally, since 2016, patients from whom an email address is known, were asked to fill out an online questionnaire that addresses contextual information, such as the patients’ country of origin, their parents’ country of origin and level of education.

### Patients

All patients in the period 2015–2020 with the diagnosis BV (ICPC-2 code X84 + ICD-10 code N76.0) or having received oral or vaginal metronidazole or vaginal clindamycin (ATC codes P01AB01, P01AB51, G01AF01, and G01AA10) in combination with a diagnosis in the category ‘female genitalia’ were included in this study. This way, we include all patients that the GPs have regarded as having BV, and were treated as such. Exclusion criteria were (i) patients that have been registered in their general practice for less than one year during the inclusion period to ensure enough follow-up time, and (ii) diagnosis of a malignancy of the female genitalia.

Currently, there is no standard definition for recurrent BV.^18^ For this study, we consider patients that have at least two episodes between 2015 and 2020 as having a recurrent condition. In order to make the diagnosis, Dutch guidelines state to use Amsel criteria. However, we speculate BV diagnosis in primary care is generally made based on clinical presentation and physical examination, that is, based on symptoms such as a greyish white, homogeneous discharge, a typical malodour, and the absence of other pathology (for example STI risk or vaginal atrophy). As we are investigating how BV diagnosis and management is approached in daily practice, in which the tools to make the diagnosis using Amsel criteria such as a microscope and pH strips might not be available, we include all patients that the GPs have regarded as having (recurrent) BV, regardless of diagnostic methods used to confirm the diagnosis.

### Variables

We analysed the following patient characteristics: age at time of first presentation, contact type and frequency, duration of symptoms at first contact, RFEs, and antibiotic use in the six months prior to an episode of BV. Moreover, we analysed the following comorbidities from 2010 onwards: STIs, PID, vaginal Candidiasis, cystitis and pelvic organ prolapse.

The types of contact were clustered by in-person contact with the GP in daily practice, remote consultation (e.g. contacts via telephone or email) in daily practice, and all types of contact with the GP during evening, night and weekend days.

All types of oral antibiotic prescriptions given in the 6 months prior to an episode of BV were considered, excluding the antibiotics that are prescribed for treating BV.

Since 2016, GPs in FaMe-Net register the duration of the reason for encounter when the patient contacts their GP for the first time. This duration is registered when the RFE is a symptom or disease, it is not registered when the RFE is a request for intervention, such as a request for medication.

Furthermore, we extracted the following data from the questionnaire that addresses contextual information: country of origin, country of origin of their parents and level of education.

The country of origin was clustered in three categories. If the patient as well as their parents were born in The Netherlands, they are defined as being Dutch. If the patient was born in another country, the country of origin is defined as ‘other’. Patients who were born in The Netherlands but have one or both parents that were born in another country, were categorised as ‘parent from other country’.

The level of education was clustered in three categories, ranging from low to high. Low educational level consists of patients that have never been to or finished any level of education, have been to elementary school only or have attended lower secondary education or lower vocational education. Intermediate level of education consists of patients that have attended intermediate/higher secondary education and intermediate vocational education. High level of education consists of patients that have attended higher vocational education or university.

Regarding the interventions performed by the GP, the following variables were analysed: prescribed medication, diagnostic tests performed, and referrals.

### Analyses

Categorical data were compared between the groups using Chi-square tests. Continuous data were compared between the groups using Mann–Whitney *U*-tests.

Top four reasons for encounter, duration of complaint, and the type of prescribed treatment are described using cross tabulation.

All statistical analyses were carried out in SPSS version 27.0. Results with a *P*-value of <0.05 based on two-sided testing were considered statistically significant.

## Results

### Patient characteristics

In total, 532 patients with BV were included (Table 1). Of these patients 91 (17.1%) presented with recurrent BV. No significant difference in age at time of first presentation between patients with recurrent BV and single-episode patients was found (*P* = 0.279).

**Table 1.** Age distribution, type of contact, and contact frequency with general practitioner in patients with one recorded episode of bacterial vaginosis and patients with recurrent BV (2015–2020).

Patient characteristics	Patients with single BV episode (*n* = 441)	Patients with recurrent BV (*n* = 91)	*P*-value
Age at time of first presentation			
<25	108 (24.5%)	14 (15.4%)	0.279^a^
26–35	133 (30.2%)	32 (35.2%)	
36–45	101 (22.9%)	21 (23.1%)	
46+	99 (22.4%)	24 (26.4%)	
Type of contact			
In-person consultation	557 (54.4%)	252 (47.7%)	0.026^a^
Remote consultation	444 (43.4%)	267 (50.6%)	
After hour services	22 (2.2%)	9 (1.7%)	
Median number of contacts per episode per patient	2.0 (IQR 1.0–3.0)	2.0 (IQR 1.5–2.6)	0.048^b^

^a^ *χ* ^2^-test.

^b^Mann–Whitney *U*-test.

The included patients had a total of 1,551 contacts with their GP across 677 episodes. Type of contact differed between both groups, as patients with recurrent BV had more remote consultation and less in-person consultation than single-episode patients (*P* = 0.026). Patients with recurrent BV contacted their GP slightly more often per BV episode (*P* = 0.048).

A duration of complaints was registered for 214 patients, of which 27 had recurrent BV and 187 were single-episode patients. We found no difference in the median duration a symptom was present before a patient contacted the GP between patients with recurrent BV and the single-episode group (*P* = 0.797).

Table 2 shows that the most frequent RFE in both groups of patients was vaginal discharge. The RFE in patients with recurrent BV was more often a request for medication (17.6% versus 8.5% in the single-episode group). Patients with recurrent complaints asked for medical advice more frequently (5.6% versus 2.4%).

**Table 2.** Top four reasons for encounter in patients with one recorded episode of BV and patients with recurrent BV (2015–2020).

ICPC-2 code	Label	RFEs (*n* = 493) in patients with single BV episode
1.X14	Vaginal discharge	194 (39.4%)
2.X15	Vaginal symptom/complaint other	46 (9.3%)
3.X50	Medication/prescription/renewal/injection	42 (8.5%)
4.X84	Vaginitis/vulvitis NOS	33 (6.7%)
		315 (63.9%)^a^
ICPC-2 code	Label	RFEs (*n* = 267) in patients with recurrent BV
1.X14	Vaginal discharge	91 (34.1%)
2.X50	Medication/prescription/renewal/injection	47 (17.6%)
3.X84	Vaginitis/vulvitis NOS	31 (11.6%)
4.X45	Observation/health education/advice/diet	15 (5.6%)
		184 (68.9%)^a^

^a^Distribution of RFEs among 677 episodes, multiple RFEs per episode is possible.

We found no significant difference in country of origin between patients with recurrent BV and single-episode patients (*P* = 0.571). There was also no significant difference in education level between the groups (*P* = 0.163).

### Medical history

Patients with recurrent BV were significantly more often prescribed at least one course of antibiotics (other than for BV) in the 6 months prior to an episode (respectively 26.4% versus 14.7% in the single-episode group, *P* = 0.007). Correcting for the number of episodes per patient, patients with recurrent BV have more episodes preceded by antibiotic use (*P* = 0.043).

Table 3 shows that patients with recurrent BV more often had at least one episode of STI (33.0% versus 18.1%, *P* = 0.001). Especially, infections with HPV were more prevalent in this group. Furthermore, Candidiasis was more frequently observed in this group as well (*P* = 0.000).

**Table 3.** Comorbidities of patients with one recorded episode of BV and patients with recurrent BV (2010–2020).

Comorbidities	Patients with single BV episode (*n* = 441)	Patients with recurrent BV (*n* = 91)	*P*-value^a^
Prolapse	17 (3.9%)	4 (4.4%)	0.809
Cystitis	242 (54.9%)	54 (59.3%)	0.435
Candidiasis	154 (34.9%)	54 (59.3%)	0.000^#^
STIs	80 (18.1%)	30 (33.0%)	0.001^#^
Chlamydia	30 (6.8%)	6 (6.6%)	0.942
Gonorrhoea	2 (0.5%)	1 (1.1%)	0.454
Trichomonas	13 (2.9%)	2 (2.2%)	0.694
Herpes genitalis	12 (2.7%)	4 (4.4%)	0.394
Condylomata acuminata	13 (2.9%)	8 (8.8%)	0.009^#^
HPV positive PAP smear	23 (5.2%)	13 (14.3%)	0.002^#^
PID	12 (2.7%)	3 (3.3%)	0.763

^a^ *χ* ^2^-test.

^#^Shows significance (*P* < 0.05) between groups.

### Interventions by GP

GPs significantly more often ordered microbiological testing in patients with recurrent BV than in patients with a single episode of BV (62.6% versus 42.6%, *P* = 0.000). Tests that can be done in the practice, such as measuring the pH of the discharge and the ‘whiff test’, were registered only twice.

Patients received a total of 745 prescriptions of either oral or vaginal antibiotics (Table 4). Oral metronidazole was most frequently prescribed, making up 87.7% and 78.8% of total prescriptions in recurrent-episode group and the single-episode, respectively. The number of prescriptions per episode was significantly higher in patients with recurrent complaints (*P* = 0.001). The recurrent-episode group also received medication significantly more often than the single-episode group (*P* = 0.000).

**Table 4.** Medication prescribed to patients with one recorded episode of BV and patients with recurrent BV (2015–2020).

Medication	Patients with single BV episode *n* = 441	Patients with recurrent BV *n* = 91	*P*-value
Antibiotic prescription			
Metronidazole (oral)	356 (78.8%)	257 (87.7%)	
Metronidazole (vaginal)	80 (18.7%)	28 (9.6%)	
Clindamycin (vaginal)	16 (3.5%)	8 (2.7%)	
Median number of prescriptions given per episode per patient	1.00 (IQR 1.00–1.00)	1.00 (IQR 1.00–1.50)	0.001 ^a^
Standard medication prescribed			
Yes	367 (83.2%)	90 (98.9%)	0.000 ^b^
No	74 (16.8%)	1 (1.1%)	

^a^Mann–Whitney *U*-test.

^b^ *χ* ^2^-test.

In total, 30 patients were referred to a gynaecologist, of which eight patients were referred twice. Patients with recurrent BV were referred more frequently than patients with single episodes of BV (11.0% versus 4.5%, *P* = 0.015).

## Discussion

To our knowledge, this is the first study that investigate the differences between patients with recurrent BV and patients with one episode of BV in a primary care population and studied GPs’ approach to patients with recurrent BV. We were able to describe the characteristics of patients that present with recurrent BV as well as identify possible risk factors for the development of recurrence.

Currently, the precise aetiology of BV is not fully elucidated. BV is known to be caused by a vaginal dysbiosis, in which opportunistic, mostly anaerobic bacteria, such as *Gardnerella vaginalis*, overgrow in lieu of healthy, protective micro-organisms, like *Lactobacillus* spp.^18^ This leads to symptoms such as a greyish white, homogeneous discharge and a typical malodour (‘fishy odour’).

In terms of possible risk factors, we found that patients of all age categories, who were recently prescribed antibiotics, and had a medical history of STIs, HPV, and/or Candidiasis significantly more often presented with recurrent BV.

Regarding the age at time of first presentation, we found similar frequencies in the four categories for both groups. This is not in line with previous findings, as BV is commonly diagnosed in patients during their reproductive years and less prevalent in persons over 40 years old, specifically postmenopausal people.^5,22^ This difference can be due to the fact that other studies were not done in a general practice population. Participants in those studies were recruited, whereas in our study we investigated presented morbidity, which might have resulted in an over-representation of postmenopausal patients in our study, as these patients might be more likely to contact their GP when suffering of abnormal discharge.

It is known that the use of antibiotics can alter a person’s microbiome, including the bacterial composition of the vaginal flora. Certain broad-spectrum penicillins, tetracyclines and nitrofuran derivatives, for example, are shown to affect *Lactobacillus* species,^23^ which are known to exert a protective effect in the vagina.^18,24^ One study has found that recent antibiotic use can lower Lactobacilli in isolated cultures by approximately 30%.^24^ This is a possible explanation for the dysbiotic state is able to relapse or persist in patients with recurrent BV. Currently, research into the role of antibiotic use and the development of BV is limited. The role of antibiotic use in the development of other types of vulvovaginitis, such as Candidiasis, however, has been more extensively documented, as well as which classes of antibiotics might trigger such an episode.^25^

Secondly, patients with recurrent BV received STI diagnoses more often than patients with single-episode BV. Although numbers of STI diagnoses were small, these findings are in line with previous studies that have shown that BV is associated with increased risk of STI acquisition and transmission.^9,10^ This facilitation of STI acquisition may be caused by the decreased protection by Lactobacilli or other lactic-acid producing micro-organisms. It can also be due to higher levels of local inflammatory cytokine production that are associated with BV.^9,26^ It is also possible that patients had more sexual partners, which is both a risk factor for BV as for STI acquisition.^27–29^

Moreover, infections with HPV were more prevalent for the group with recurrent BV as well, which is in line with previous research. Gilet and colleagues^30^ conducted a meta-analysis, which showed a positive association between BV and HPV. Research also shows that patients with healthy vaginal flora, dominated by *Lactobacillus* spp., achieved higher clearance rates of HPV.^31^

Candida diagnoses were also more prevalent for the group with recurrent BV. This can have several possible explanations. For one, diagnoses can be overestimated because the recurrent group received more microbiological testing in which Candida could be detected. Patients could also suffer from mixed infections, where Candida and BV are present simultaneously.^32^ Furthermore, BV and medication for BV, such as metronidazole and clindamycin have been described as risk factors for the development of vulvovaginal Candidiasis. Lastly, the group with recurrent BV received more antibiotic prescriptions, which is also a known risk factor for Candidiasis.^25^

We found that patients with recurrent BV had more remote consultations and less in-person consultations than single-episode patients and the RFE was more often a request for medication. This can be explained by patients’ recognition of the symptoms from previous episodes. A qualitative study by Payne and colleagues^33^ shows patients with recurrent BV would like to have an over-the-counter treatment available and the majority would feel comfortable self-medicating.

With regard to the GPs’ approach, although microbiological tests were more frequently performed for patients with recurrent BV than for single-episode patients, diagnostic testing was not performed in more than 37% of the recurrent-episode patients, even though Dutch guidelines state to use Amsel criteria to make the diagnosis.^17^ We speculate that the diagnosis was based on the clinical presentation and physical examination.

Moreover, the majority of patients in both groups were prescribed medication with oral metronidazole most frequently prescribed. Although metronidazole is considered treatment of first choice, one study has shown that 68% of isolated strains of *Gardnerella vaginalis* are resistant to metronidazole which highlights the need for better treatment options.^34^

### Limitations

One of the strengths of this study is that the validity of registration is high, as participating GPs code all information in a systematic manner and have regular meetings to uphold the quality of registration. The results of this study are representative for the general Dutch population. Additionally, because participating GPs register all encounters and all interventions performed during an episode of care, it is possible to evaluate the GPs actions as well as researching the patient characteristics. On the other hand, this means that we can not evaluate patient compliance.

Another limitation of our study is that the sample sizes of the patients with STIs are relatively small. Studies into the association between STI acquisition and BV often encompass high-risk populations, such as sex workers, whereas our study population is representative for the general Dutch population, in which incidence of STIs is lower. At the same time, this is one of the strengths of this study, as the results are not limited to a subgroup of patients. It is also possible that the STI and Candida diagnoses are overestimated in the group of recurrent BV patients, because they have received more microbiological tests than the single-episode group. This may lead to more incidental findings and possibly does not accurately reflect the actual numbers of diagnoses.

## Conclusion

This study adds to the knowledge on the dismal effect of antibiotic use on vaginal dysbiosis. We suggest medical professionals take this into account when counselling patients known to have recurrent BV when they need antibiotics for another condition.

Furthermore, we suggest that GPs prioritise in-person consultation over repeat prescriptions in patients with recurrent complaints. Critical evaluation of the (previous) diagnosis is justified using standardised criteria, such as Amsel criteria, and warrants microbiological testing before treatment is installed again, to prevent unnecessary prescriptions and further development of antibiotic resistance.

In conclusion, the present study provides tools for GPs to better recognise patients at risk of developing recurrent complaints and re-evaluate their approach to the diagnosis and treatment of recurrent BV.

## Supplementary Material

cmad005_suppl_Supplementary_Material

## Data Availability

Data generated at a large-scale facility (available upon request). The data underlying this article were accessed from FaMe-Net (https://www.famenet.nl/), a primary care registration network connected to RadboudUMC.
